# Renovascular hypertension increases serum TNF and CX3CL1 in experimental *Trypanosoma cruzi* infection

**DOI:** 10.1590/1414-431X20186690

**Published:** 2018-03-26

**Authors:** M.C. Silva, M.A. Azevedo, V.P. Figueiredo, M.R. Moura, D. Coelho, P.M. Martinelli, R.P. Machado, A.C. Alzamora, A. Talvani

**Affiliations:** 1 Departamento de Ciências Biológicas, Universidade Federal de Ouro Preto, Ouro Preto, MG, Brasil; 2 Escola de Medicina, Universidade Federal de Ouro Preto, Ouro Preto, MG, Brasil; 3 Programa de Pós-Graduação em Ciências Biológicas, Universidade Federal de Ouro Preto, Ouro Preto, MG, Brasil; 4 Programa de Pós-Graduação em Saúde e Nutrição, Universidade Federal de Ouro Preto, Ouro Preto, MG, Brasil; 5 Programa de Pós-Graduação em Ecologia e Biomas Tropicais, Universidade Federal de Ouro Preto, Ouro Preto, MG, Brasil; 6 Departamento de Morfologia, Universidade Federal de Minas Gerais, Belo Horizonte, MG, Brasil

**Keywords:** Trypanosoma cruzi, Hypertension, Tumor necrosis factor, CX3CL1, Cardiac inflammation

## Abstract

*Trypanosoma cruzi* triggers a progressive inflammatory response affecting cardiovascular functions in humans and experimental models. Angiotensin II, a key effector of the renin-angiotensin system, plays roles in mediating hypertension, heart failure, and inflammatory responses. *T. cruzi* and AngII can induce inflammatory responses by releasing inflammatory mediators. The aim of this study was to evaluate systemic AngII, tumor necrosis factor (TNF), and CX3CL1 mediators in a two-kidney one-clip (2K1C) renovascular hypertension model using Wistar rats infected with *T. cruzi*. Our data showed an increase in serum AngII in uninfected and *T. cruzi-*infected rats 1 week after 2K1C surgery compared to non-2K1C (Sham) animals. The baseline systolic blood pressure was higher in both uninfected and infected 2K1C rats. Despite no difference in circulating parasites in the acute phase of infection, elevated serum TNF and CX3CL1 were observed at 8 weeks post-infection in 2K1C rats in association with higher cardiac inflammatory infiltration. In summary, AngII-induced hypertension associated with *T. cruzi* infection may act synergistically to increase TNF and CX3CL1 in the 2K1C rat model, thereby intensifying cardiac inflammatory infiltration and worsening the underlying inflammation triggered by this protozoan.

## Introduction

*Trypanosoma cruzi* is a flagellated protozoan whose membrane glycoproteins act as antigens capable of triggering an inflammatory response in mammalian hosts (1). This immune-cell activation promotes the release of inflammatory mediators and antibodies that regulate parasite entry into the bloodstream. However, because of its well-adapted escape mechanisms, the immune system typically fails and *T. cruzi* persists in muscle cells. Once these parasites reach the cardiac cells, they trigger a local and persistent inflammatory response that is inevitably associated with cellular and tissue destruction (2).

Both human Chagas disease and experimental models of *T. cruzi* infection not only affect heart muscle, but also promote endothelial dysfunction of systemic blood vessels (3,4). Thus, hypertension is a common condition affecting the cardiovascular system and the functioning of other physiological systems (5). Hyper-activity of the renin-angiotensin system (RAS), particularly angiotensin II (AngII), together with sympathetic nervous system over-activity contribute to the development and maintenance of hypertension. Moreover, AngII promotes cardiac remodeling by stimulating inflammatory processes, cardiomyocyte hypertrophy, and fibroblast proliferation. This may eventually lead to cardiac fibrosis, which not only affects cardiac function, but also the general health of the individual (6).

The two-kidney, one-clip (2K1C) renovascular-hypertension model has been widely used to induce overproduction of RAS (7). The time-course of this hypertensive model presents different stages after clipping of the renal artery. In the early phase (approximately 4 weeks after clipping), high blood pressure is induced by plasma renin activity and circulating AngII. In the later phase (after 8 weeks), the plasma renin activity and AngII levels are reduced, but the hypertension is maintained (8,9).

Because both hypertension and *T. cruzi* activate inflammatory mediators, we hypothesized that both clinical conditions act synergistically to worsen local and systemic pathological effects by inducing and maintaining a long-term inflammatory response. In this study, we investigated the production of serum tumor necrosis factor (TNF), CX3CL1, and other physiological parameters in *T. cruzi*-infected hypertensive Wistar rats.

## Material and Methods

### Animals

Male Wistar rats weighing approximately 180–200 g were bred and maintained at the animal facility at Universidade Federal de Ouro Preto (UFOP), Ouro Preto, MG, Brazil. The animals were maintained in cages under standard conditions and given *ad libitum* access to food and water. All procedures were followed in accordance with the guidelines issued by the Brazilian College of Animal Experimentation and approved by the Ethical Committee for Experiments with Laboratory Animals at UFOP (Protocol# 2011/44).

### Induction of renovascular hypertension

2K1C renovascular-hypertension was induced as described previously (10). Briefly, after a 24-h fast, a group of 8 rats (150–200 g) was anesthetized with a combination of ketamine and xylazine (80 and 10 mg/kg, intra-peritoneal, respectively). A U-shaped silver clip (8 mm in length and 2 mm in width) with a 0.2-mm internal diameter was placed around the left renal artery through a mid-line incision. The control group of animals (Sham group or normotensive rats) was subjected to a similar procedure, except for placement of the renal artery clip. This procedure generated two different groups: hypertensive rats (2K1C surgery group) and normotensive rats (Sham group).

### Experimental infection

Parasitological infection was conducted using the trypomastigote-form of the Y- strain of *T. cruzi*. The bloodstream trypomastigote form of the parasite was maintained in Swiss mice by serial passage of infected blood (11). Immediately after 2K1C surgery, rats were intraperitoneally inoculated, with 1.2×10^6^ trypomastigotes obtained from previously infected Swiss mice (12). Control mice were injected only with the vehicle, phosphate-buffered saline. This procedure generated two different groups of rats: an infected and non-infected group.

Parasitemia was determined every day by direct observation using an optical microscope. To quantify bloodstream parasitemia, 5 μL of peripheral blood from infected animals was smeared on a microscope slide (22×22 mm), and the number of parasite trypomastigotes was determined by counting 100 fields as described by Brener in 1962 (13).

### Blood pressure measurement

Systolic arterial blood pressure was measured every week in all groups of rats by the non-invasive tail-cuff plethysmography method using the Panlab non-invasive blood pressure system for rodents (Harvard Apparatus, UK). After pre-conditioning, rats were subjected to heat treatment at 35±2°C in a plastic restrainer and a cuff with a pneumatic pulse sensor was attached to the tail. The individual measurements of blood pressure were calculated as the mean of 15 measurements.

### Immunoassay

Peripheral blood was collected by cardiac puncture at 1 and 8 weeks post-2K1C surgery and *T. cruzi* infection. After centrifugation at 3000 *g* for 10 min at 4°C, serum was collected and Fractalkine/CX3CL1 (R&D Systems, USA) and TNF (PeproTech, USA) levels were evaluated by immunoassay (ELISA) using a kit as per the manufacturer's instructions. Briefly, rat serum samples and the protein of interest (or standard) were added to a 96-well plate that was pre-coated with a monoclonal antibody specific for rat Fractalkine/CX3CL1 or TNF. After 1 h of incubation at 20°C, plates were washed and incubated with detection antibody for 30 min. The plates were washed again and incubated with 3,3′,5,5′-tetramethylbenzidine (TMB) substrate for 15 min. After the reaction stopped, the absorbance at 450 nm was measured using a microplate reader (Bio-Rad, USA).

### Angiotensin II quantification assay

The rat serum AngII level was determined using an ELISA assay kit (Wuhan USCN Business Co., Ltd., China) at 1 and 8 weeks after 2K1C surgery and *T. cruzi* infection. The assay was performed according to the manufacturer's instructions. Briefly, peripheral blood was collected through cardiac puncture. Following centrifugation at 3000 *g* for 10 min at 4°C, serum was collected and added to the appropriate wells of a 96-well plate that was pre-coated with a monoclonal antibody specific for rat AngII. After 1 h of incubation at 37°C, the plate was washed and incubated with detection antibody for 30 min. After washing, the plate was incubated with TMB substrate for 15 min. The reaction was stopped and absorbance at 450 nm was measured in a microplate reader (Bio-Rad). The concentrations used for plotting the ELISA standard curve were 1000, 333.33, 111.11, 37.04, and 12.35 pg/mL. The limit of sensitivity for the assay was 5.92 pg/mL.

### Histological analysis

After 8 weeks of infection, groups of 6 rats were sacrificed and fragments of the heart from each rat were subjected to histological analysis. Heart tissues were fixed in 10% buffered formalin solution. After 48 h, tissue was dehydrated in increasing concentration of ethanol solutions, embedded in paraffin, and cut into 5-µm thick sections. The sections were stained with hematoxylin-eosin (H&E) and Masson's trichrome to detect inflammation and collagen, respectively, using the Image J 1.45s program (National Institutes of Health, USA). Twenty random fields (74,931 µm^2^ each) from each H&E or Masson's trichrome-stained sections were analyzed at 40× magnification, resulting in a total of 1.49×10^6^ µm^2^ of analyzed myocardium area. Images were captured using a Leica DM 5000 B microchamber (Leica Application Suite, Germany, version 2.4.0 R1). Total cell nuclei (including nuclei from leucocytes and cardiac cells) were counted in the heart tissues of non-infected, infected, Sham, and 2K1C rats. The degree of inflammation was calculated as the number of nuclei in the infected group divided by the corresponding number in the non-infected group, as previously described by Caldas et al. (14). Fibrosis was quantified based on the total area occupied by collagen.

### Statistical analysis

Data are reported as means±SE. Statistical analysis was performed by one-way analysis of variance (ANOVA) followed by the Bonferroni post-test or Student's *t*-test for unpaired data, as appropriate. For all analyses, Graph Pad Prism v.6 (Graph Pad Software, USA) was used. Differences were considered to be statistically significant at a P-value less than 0.05.

## Results

### Blood parasitemia and baseline systolic blood pressure

**Figure 1.**
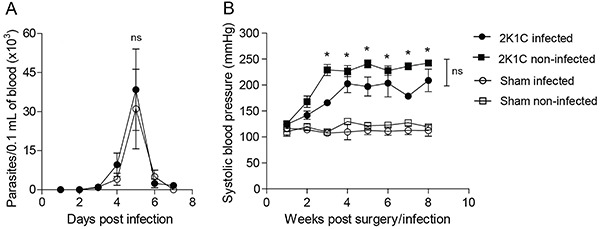
Parasitemia and systolic blood pressure. Wistar rats were subjected to two-kidney, one-clip (2K1C) (n=8) renovascular hypertension or SHAM normotensive (n=8) surgery and infected or not with 1.2×10^6^ trypomastigote forms of *T. cruzi* Y strain. Parasitemia was measured daily (*A*) and systolic blood pressure was measured weekly (*B*). Data are reported as means±SE. *P<0.05 compared with the Sham groups (two-way ANOVA). ns: not significant.

Although arterial hypertension may positively modulate inflammation, which in turn controls circulating parasites, we observed no significant difference between parasitemia of 2K1C rats and Sham animals (Figure 1A). Even when the rats were infected 3 weeks after 2K1C surgery during the period when the blood pressure is already high, blood parasitemia was similar in both the 2K1C and Sham groups (data not shown). Next, we assessed whether *T. cruzi* infection could modulate arterial hypertension after 2K1C surgery. The baseline values of systolic blood pressure of non-infected and infected 2K1C rats were higher (P<0.05) than in Sham rats, at 3 and 8 weeks after surgery. Additionally, *T. cruzi* infection did not modulate the baseline values of systolic blood pressure in 2K1C rats (Figure 1B).

### Serum AngII levels

**Figure 2.**
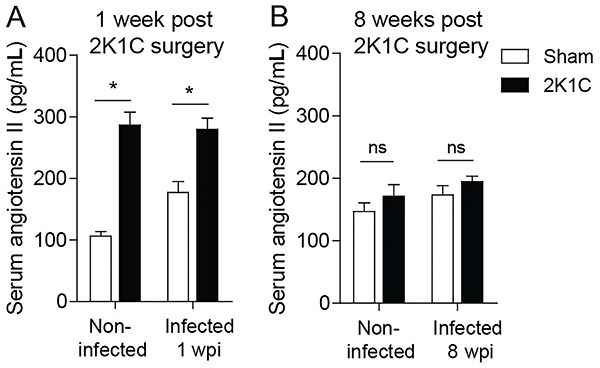
Serum AngII in two-kidney one-clip (2K1C) infected rats. Serum angiotensin II was measured in Wistar rats by immunoassay at 1 (*A*) and 8 (*B*) weeks after the 2K1C renovascular (n=8) and Sham (n=8) surgeries, immediately followed or not by infection with 1.2×10^6^ trypomastigote forms of Y strain of the *T. cruzi*. wpi: weeks post-infection. Data are reported as means±SE. *P<0.05, one-way ANOVA followed by Bonferroni post-test. ns: non-significant difference. wpi: weeks post-infection.

During the initial phase of 2K1C surgery (after 1 week), serum AngII levels were higher in 2K1C rats (black bars) than in Sham rats (white bars). The parasite infection did not affect serum AngII levels (Figure 2A). However, in the late stage of 2K1C surgery (after 8 weeks), AngII returned to basal levels in hypertensive rats (black bars) and no difference was observed between non-infected and infected groups (Figure 2B).

### Serum inflammatory mediators

**Figure 3.**
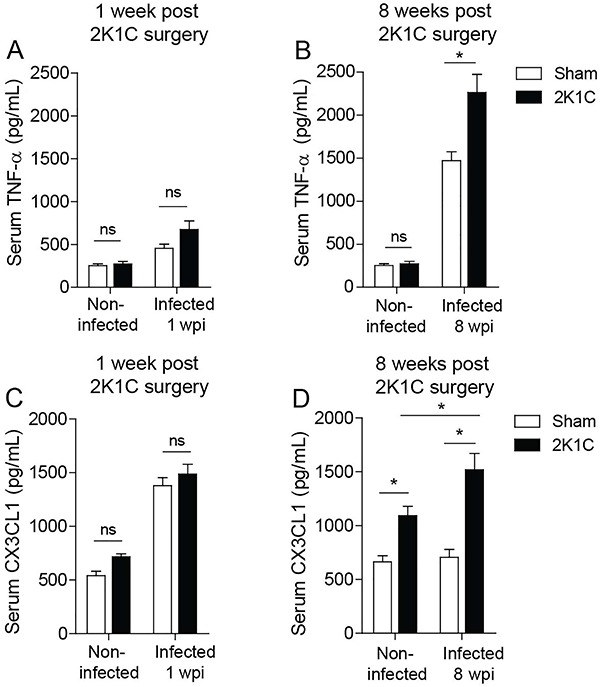
Serum TNF and CX3CL1 in infected two-kidney one-clip (2K1C) rats. One and 8 weeks after 2K1C (n=8) or Sham (n=8) surgery, immediately followed, or not, by infection with 1.2×10^6^ trypomastigote forms of the Y strain of *T. cruzi*, the serum levels of TNF (*A* and *B*) and CX3CL1/Fractalkine (*C* and *D*) were measured by ELISA assay. Data are reported as means±SE. *P<0.05, one-way ANOVA followed by Bonferroni post-test. ns: non-significant difference. wpi: weeks post-infection.

One week after 2K1C surgery, TNF production did not differ between 2K1C and Sham rats (Figure 3A); however, in the late stage of infection and surgery (after 8 weeks), TNF production was higher in hypertensive rats than in the Sham group (Figure 3B). The level of CX3CL1 increased similarly in 2K1C and Sham rats at 1 week post-infection compared to non-infected animals (Figure 3C). However, 8 weeks after surgery, both non-infected and infected 2K1C hypertensive rats showed increased levels of CX3CL1 (black bars; Figure 3D). Moreover, the infection resulted in a sustained increase in CX3CL1 levels only in 2K1C hypertensive rats, while this chemokine returned to basal levels in Sham animals (Figure 3C and D).

### Heart inflammation and collagen

**Figure 4.**
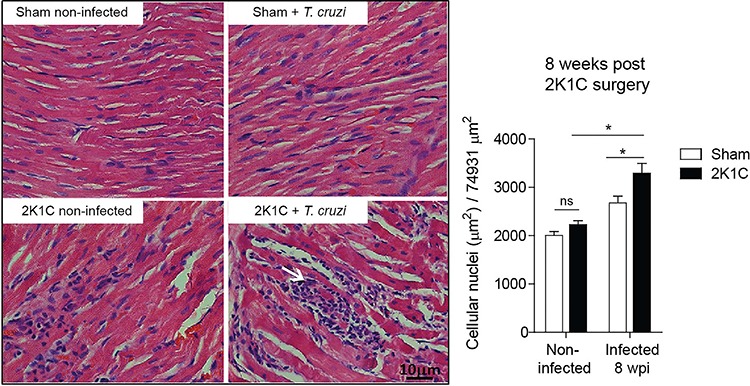
Inflammatory leukocytes in two-kidney one-clip (2K1C) infected cardiac tissue. Before and eight weeks after 2K1C (n=8) and Sham (n=8) surgeries, immediately followed, or not, by infection with the Y strain of *T. cruzi*, cardiac tissues were stained with hematoxylin and eosin to quantify cellular nuclei (from leukocytes and cardiac cell nuclei). This analysis was performed using the *ImageJ* program in a total area of 74,931 µm^2^ (magnification 40×). Data are reported as means±SE. *P≤0.05, one-way ANOVA followed by Bonferroni post-test. ns: non-significant difference. White arrow: leukocyte infiltration. wpi: weeks post-infection. Magnification bar: 10 μm.

**Figure 5.**
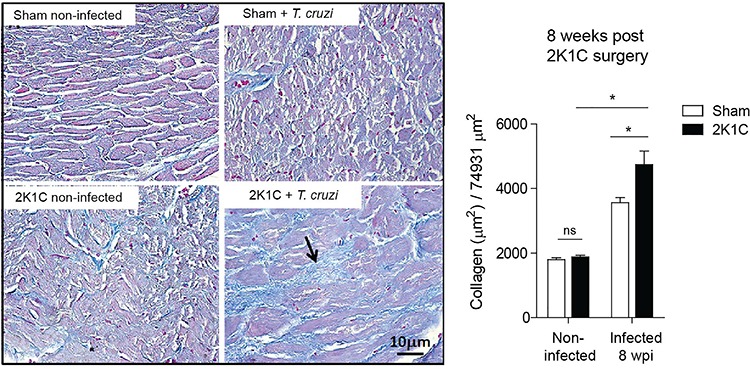
Collagen analysis in two-kidney one-clip (2K1C) infected cardiac tissue. Before and 8 weeks after 2K1C (n=8) and Sham (n=8) surgeries, immediately followed, or not, by infection with the Y strain of *T. cruzi*, cardiac tissues were stained with Masson's trichrome to quantify collagen using the *ImageJ* program in a total area of 74,931 µm^2^ (magnification 40×). Data are reported as means±SE. *P≤0.05 by one-way ANOVA followed by Bonferroni post-test. ns: non-significant difference. Black arrow: collagen. wpi: weeks post-infection. Magnification bar: 10 μm.

Because infection with the Y-strain of *T. cruzi* is characterized by intense infiltration of inflammatory cells into the rodent cardiac muscle, we also evaluated whether 2K1C renovascular-hypertension would impact heart tissue inflammation at 8 weeks post-infection. Interestingly, although *T. cruzi* did not induce heart inflammation in Sham rats, we found an increased number of inflammatory cells in the heart tissue of 2K1C hypertensive rats at 8 weeks post-infection (Figure 4). Accordingly, Masson's trichrome staining indicated that collagen expression was also elevated in rats subjected to 2K1C renovascular surgery, particularly in those infected with *T. cruzi* (Figure 5).

## Discussion

In the last 20 years, Brazilian government programs aiming to prevent humans from coming into contract with the insect vector of Chagas disease resulted in a partial reduction of seropositivity among young people. However, the prevalence remains high among adults and the elderly (15). Hypertension is a disease that progresses with advancing age and shows a high prevalence amongst Chagas patients (16–18). Therefore, we evaluated whether the co-occurrence of both diseases could interfere with the inflammatory, parasitological, and physiological pathways using the 2K1C arterial-hypertension model for Wistar rats infected with the Y-strain of *T. cruzi*.

In the present study, AngII-induced hypertension in 2K1C-infected animals did not impact the load of circulating *T. cruzi* during the initial phase of infection. The 2K1C model of hypertension is well-characterized, and it is known that sustained hypertension in the later phase is AngII-independent, as elevated AngII is not exclusively responsible for its maintenance (18). The time course of the 2K1C model is divided into phases after clipping of the renal artery: i) 4 weeks: blood pressure increases, which is associated with increases in plasma renin activity and circulating AngII concentration; ii) 5–8 weeks: hypertension associated with increasing RAS components in tissue, despite reduced plasma renin activity and circulating AngII; iii) 9 weeks and later: hypertension is maintained by increased activity of tissue RAS at the level of plasma volume and in a sympathetic manner. Our results are in accordance with those data, as we found higher circulating levels of AngII in 2R1C rats in the first week after surgery and similar levels between 2R1C and Sham rats at 8 weeks. There is an interplay between increased peptides hormones from the kallikrein-kinin system (e.g., kinins), which reduces AngII and blood pressure during acute and chronic events (19). *T. cruzi* activates the kinin pathway through its major cysteine proteinase, which is associated with parasite invasion and the development of tissue inflammation (20). Although excessive activation of the kinin system may promote worsening of vascular system function, the kinins pathway is fine-tuned with overlapping internal regulatory mechanisms in mammals. In this study, we hypothesized that *T. cruzi* may induce overproduction of kinins during long-term infection (e.g., 8 weeks after preparing the 2K1C model of hypertension), thereby contributing to plasma control of AngII release.

Although serum AngII returns to its basal levels in the later stage of *T. cruzi* infection, renovascular-hypertension continues to inhibit TNF production. Previous studies showed that TNF is elevated during hypertension in patients as well as in experimental models, and blockage of the TNF receptor prevents hypertension development in rats (10,21). Earlier studies also demonstrated activation and elevated leukocyte counts in hypertensive rats compared to normotensive animals (22). Thus, hypertension is regarded as chronic low-grade inflammation with elevated levels of pro-inflammatory mediators in patients and experimental models. Similarly, there is a strong association between *T. cruzi* infection and up-regulation of TNF in serum and tissues (23,24). This inflammatory cytokine is produced mainly by mononuclear cells upon primary activation by *T. cruzi* glycoproteins and other antigens. Subsequently, the paracrine activity of TNF exacerbates the inflammatory response and affects distinct metabolic pathways as part of *T. cruzi*-induced cardiovascular pathogenesis (25–27 [Bibr B28]). TNF is associated with disease progression to cardiac failure in chagasic patients, and genetic polymorphisms in this gene may be associated with protection during infection (27–29). In the *T. cruzi* infection model, AngII-induced hypertension and TNF up-regulation may contribute to macrophages (M-1 and M-2)-mediated inflammation in a TNF-dependent pathway that is activated during fibrosis development and consequent remodeling of the cardiac tissue.

Inflammatory cytokines and chemokines can induce expression of chemotactic cytokine CX3CL1, by monocytes/macrophages, endothelial, and smooth muscle cells. As CX3CL1 binds and signals through the CX3CR1 receptor, once this chemokine is released, it shows chemotaxis towards CX3CR1-expressing inflammatory cells, such as macrophages and T and NK cells, into the tissues (30). AngII has been shown to up-regulate CX3CR1 receptor expression in a human monocyte cell line derived from acute monocytic leukemia patients, indicating its potential regulatory role in blood mononuclear cell activation and migration to inflammation sites (31). This CX3CR1 up-regulation may promote firm adhesion of circulating CX3CR1-positive cells to CXC3L1-expressing endothelial cells, thereby inducing vascular inflammation and amplification of the local inflammatory response. Because of its role in inflammatory pathways, CX3CL1 is recognized as an independent predictor of mortality among advanced heart failure patients. Indeed, circulating levels were lower in individuals administered angiotensin-converting enzyme inhibitor-therapy (32). These results further reinforce the correlation between CX3CL1 and the pathogenesis of hypertension/cardiovascular diseases.

In addition to the above described role, some studies have shown that CX3CL1 and AngII promote phosphorylation of mitogen-activated protein kinases and serine-threonine kinases (33,34). This hypothesis is supported by the reported association between mitogen-activated protein kinases and molecular events leading to fibrosis (35,36). Fibrosis induced by inflammatory events is an important step in organ remodeling that is associated with clinical manifestation of *T. cruzi* infection (37). Thus, CX3CL1 induced by hypertensive-endothelial cell injury may promote remodeling of healthy organs and potentiate the recovery of previously damaged organs.

A few studies have demonstrated the involvement of CX3CL1 in *T. cruzi* pathogenesis. It is known that during experimental *T. cruzi* infection, CD8^+^ and CD4^+^ T cells express the receptor of CX3CL1, and high serum levels of CX3CL1 in male Fischer rats was previously observed in the initial phase of *T. cruzi* infection (38,39). Interestingly, when infected animals were fed with a low-protein diet in the acute or chronic phase of infection, CX3CL1 levels in the serum and cardiac homogenate were elevated. Thus, the AngII-dependent inflammatory process is regulated by TNF, CX3CL1, and other inflammatory mediators (IL-12, IFN-γ, PAF, CCL2-5, and others) in distinct etiologies (40). This pathway may be critical for promoting myocardial damage during *T. cruzi* infection, as inflammation is the origin of this illness.

In this study, we showed that AngII-induced renovascular hypertension up-regulated TNF and CX3CL1 production in *T. cruzi*-infected rats, thereby further potentiating cardiac inflammation and fibrosis. In this context, the hypertensive condition may aggravate the immuno-pathogenesis of *T. cruzi* infection.

## References

[1] Ropert C, Ferreira LR, Campos MA, Procopio DO, Travassos LR, Ferguson MA (2002). Macrophage signaling by glycosylphosphatidylinositol-anchored mucin-like glycoproteins derived from *Trypanosoma cruzi* trypomastigotes. Microbes Infect.

[2] Talvani A, Teixeira MM (2011). Inflammation and Chagas disease some mechanisms and relevance. Adv Parasitol.

[3] Rossi MA (1995). Pathogenesis of chronic Chagas' myocarditis. São Paulo Med J.

[4] Camandaroba E, The TS, Pessina DH, Andrade SG (2006). *Trypanosoma cruzi*: clones isolated from the Colombian strain, reproduce the parental strain characteristics, with ubiquitous histotropism. Int J Exp Pathol.

[5] Sowers JR, Epstein M, Frohlich ED (2001). Diabetes, hypertension, and cardiovascular disease: an update. Hypertension.

[6] Gonzalez A, Lopez B, Querejeta R, Diez J (2002). Regulation of myocardial fibrillar collagen by angiotensin II. A role in hypertensive heart disease?. J Mol Cell Cardiol.

[7] Okamura T, Miyazaki M, Inagami T, Toda N (1986). Vascular renin-angiotensin system in two-kidney, one clip hypertensive rats. Hypertension.

[8] Amiri F, Garcia R (1997). Renal angiotensin II receptor regulation in two-kidney, one clip hypertensive rats: effect of ACE inhibition. Hypertension.

[9] Maia RC, Sousa LE, Santos RA, Silva ME, Lima WG, Campagnole-Santos MJ (2015). Time-course effects of aerobic exercise training on cardiovascular and renal parameters in 2K1C renovascular hypertensive rats. Braz J Med Biol Res.

[10] Goldblatt H, Lynch J, Hanzal RF, Summerville WW (1934). Studies on experimental hypertension: I. The production of persistent elevation of systolic blood pressure by means of renal ischemia. J Exp Med.

[11] Lalonde RG, Holbein BE (1984). Role of iron in *Trypanosoma cruzi* infection of mice. J Clin Invest.

[12] Carneiro ZA, Maia PI, Sesti-Costa R, Lopes CD, Pereira TA, Milanezi CM (2014). *In vitro* and *in vivo* trypanocidal activity of H2bdtc-loaded solid lipid nanoparticles. PLoS Negl Trop Dis.

[13] Brener Z (1962). Therapeutic activity and criterion of cure on mice experimentally infected with *Trypanosoma cruzi*. Rev Inst Med Trop São Paulo.

[14] Caldas IS, Talvani A, Caldas S, Carneiro CM, de Lana M, da Matta Guedes PM (2008). Benznidazole therapy during acute phase of Chagas disease reduces parasite load but does not prevent chronic cardiac lesions. Parasitol Res.

[15] Coura JR (2007). Chagas disease: what is known and what is needed - a background article. Mem Inst Oswaldo Cruz.

[16] Moncayo A, Ortiz Yanine MI (2006). An update on Chagas disease (human American trypanosomiasis). Ann Trop Med Parasitol.

[B17] Coura JR, Borges-Pereira J (2010). Chagas disease: 100 years after its discovery. A systemic review. Acta Trop.

[18] Martinez-Maldonado M, Benabe JE (1987). Hypertension in renovascular disease. Contrib Nephrol.

[19] Golias Ch, Charalabopoulos A, Stagikas D, Charalabopoulos K, Batistatou A (2007). The kinin system-bradykinin: biological effects and clinical implications. Multiple role of the kinin system-bradykinin. Hippokratia.

[20] Scharfstein J, Schmitz V, Morandi V, Capella MM, Lima AP, Morrot A, Juliano L, Müller-Esterl W (2000). Host cell invasion by *Trypanosoma cruzi* is potentiated by activation of bradykinin B(2) receptors. J Exp Med.

[21] Brody MJ, Varner KJ, Vasquez EC, Lewis SJ (1991). Central nervous system and the pathogenesis of hypertension. Sites and mechanisms. Hypertension.

[22] Schmid-Schonbein GW, Seiffge D, DeLano FA, Shen K, Zweifach BW (1991). Leukocyte counts and activation in spontaneously hypertensive and normotensive rats. Hypertension.

[23] Derouich-Guergour D, Brenier-Pinchart MP, Ambroise-Thomas P, Pelloux H (2001). Tumour necrosis factor alpha receptors: role in the physiopathology of protozoan parasite infections. Int J Parasitol.

[24] Vasconcelos RH, Azevedo Ede A, Diniz GT, Cavalcanti Mda G, de Oliveira W J, de Morais CN (2015). Interleukin-10 and tumour necrosis factor-alpha serum levels in chronic Chagas disease patients. Parasite Immunol.

[25] Lima ES, Andrade ZA, Andrade SG (2001). TNF-alpha is expressed at sites of parasite and tissue destruction in the spleen of mice acutely infected with *Trypanosoma cruzi*. Int J Exp Pathol.

[B26] Aliberti JC, Souto JT, Marino AP, Lannes-Vieira J, Teixeira MM, Farber J (2001). Modulation of chemokine production and inflammatory responses in interferon-gamma- and tumor necrosis factor-R1-deficient mice during *Trypanosoma cruzi* infection. Am J Pathol.

[27] Drigo SA, Cunha-Neto E, Ianni B, Cardoso MR, Braga PE, Fae KC (2006). TNF gene polymorphisms are associated with reduced survival in severe Chagas' disease cardiomyopathy patients. Microbes Infect.

[B28] Cunha-Neto E, Nogueira LG, Teixeira PC, Ramasawmy R, Drigo SA, Goldberg AC (2009). Immunological and non-immunological effects of cytokines and chemokines in the pathogenesis of chronic Chagas disease cardiomyopathy. Mem Inst Oswaldo Cruz.

[29] Pissetti CW, Correia D, de Oliveira RF, Llaguno MM, Balarin MA, Silva-Grecco RL (2011). Genetic and functional role of TNF-alpha in the development *Trypanosoma cruzi* infection. PLoS Negl Trop Dis.

[30] Apostolakis S, Spandidos D (2013). Chemokines and atherosclerosis: focus on the CX3CL1/CX3CR1 pathway. Acta Pharmacol Sin.

[31] Apostolakis S, Vlata Z, Vogiatzi K, Krambovitis E, Spandidos DA (2010). Angiotensin II up-regulates CX3CR1 expression in THP-1 monocytes: impact on vascular inflammation and atherogenesis. J Thromb Thrombolysis.

[32] Richter B, Koller L, Hohensinner PJ, Rychli K, Zorn G, Goliasch G (2012). Fractalkine is an independent predictor of mortality in patients with advanced heart failure. Thromb Haemost.

[33] Klosowska K, Volin MV, Huynh N, Chong KK, Halloran MM, Woods JM (2009). Fractalkine functions as a chemoattractant for osteoarthritis synovial fibroblasts and stimulates phosphorylation of mitogen-activated protein kinases and Akt. Clin Exp Immunol.

[34] Zhuo JL, Kobori H, Li XC, Satou R, Katsurada A, Navar LG (2016). Augmentation of angiotensinogen expression in the proximal tubule by intracellular angiotensin II via AT1a/MAPK/NF-small ka, CyrillicB signaling pathways. Am J Physiol Renal Physiol.

[35] Wang S, Ding L, Ji H, Xu Z, Liu Q, Zheng Y (2016). The role of p38 MAPK in the development of diabetic cardiomyopathy. Int J Mol Sci.

[36] Miao R, Lu Y, Xing X, Li Y, Huang Z, Zhong H (2016). Regulator of G-protein signaling 10 negatively regulates cardiac remodeling by blocking mitogen-activated protein kinase-extracellular signal-regulated protein kinase 1/2 signaling. Hypertension.

[37] Rodriguez-Zanella H, Melendez-Ramirez G, Velazquez L, Meave A, Alexanderson E (2015). ECG score correlates with myocardial fibrosis assessed by magnetic resonance: A study in Chagas heart disease. Int J Cardiol.

[38] Maldonado IR, Ferreira ML, Camargos ER, Chiari E, Machado CR (2004). Skeletal muscle regeneration and *Trypanosoma cruzi*-induced myositis in rats. Histol Histopathol.

[39] Martins RF, Martinelli PM, Guedes PM, da Cruz Pádua B, Dos Santos FM, Silva ME (2013). Protein deficiency alters CX3CL1 and endothelin-1 in experimental *Trypanosoma cruzi*-induced cardiomyopathy. Trop Med Int Health.

[40] Munoz-Durango N, Fuentes CA, Castillo AE, Gonzalez-Gomez LM, Vecchiola A, Fardella CE (2016). Role of the renin-angiotensin-aldosterone system beyond blood pressure regulation: molecular and cellular mechanisms involved in end-organ damage during arterial hypertension. Int J Mol Sci.

